# Rehabilitating Soldiers for Load Carriage Tasks: An International Perspective

**DOI:** 10.3390/ijerph22081286

**Published:** 2025-08-17

**Authors:** Robin Orr, Joseph J. Knapik, Rachel Rodgers, Robyn Cassidy, Jacques Rousseau, Damien Van Tiggelen, Rodney Pope

**Affiliations:** 1 Faculty of Health Sciences and Medicine, Bond University, Robina, QLD 4226, Australia; rpope@csu.edu.au; 2 Tactical Research Unit, Bond University, Robina, QLD 4226, Australia; 3 Military Nutrition Division, U.S. Army Research Institute of Environmental Medicine, Natick, MA 01760, USA; joseph.j.knapik.vol@health.mil; 4 Defence Medical Rehabilitation Centre, Stanford Hall, Loughborough LE12 5QW, UK; rachel.rodgers200@mod.gov.uk (R.R.); robyn.cassidy104@mod.gov.uk (R.C.); 5 Human Performance Cell, Joint Support Group, New Zealand Army, Waiouru 4825, New Zealand; jacques.rousseau@nzdf.mil.nz; 6 Belgium Armed Forces, 1140 Brussels, Belgium; damien.vantiggelen@mil.be; 7 School of Allied Health, Exercise and Sports Sciences, Charles Sturt University, Albury, NSW 2640, Australia

**Keywords:** ruck march, tabbing, pack march, reconditioning, military

## Abstract

Soldiers are likely to suffer an injury and require rehabilitation at some stage of their career. Load carriage, whilst a fundamental requirement, is also a source of injury risk. To optimize the rehabilitation of soldiers and prepare them for a full return to operational duty, load carriage requirements need to be considered throughout their rehabilitation pathway. In addition, injury risks associated with load carriage need to be considered to inform mitigation of reinjury. During the initial injury treatment stage, loss of key fitness elements associated with load carriage performance, being aerobic fitness and relative strength, need to be minimized. Any losses of these same elements of fitness then need to be considered in the overall reconditioning stage. Finally, with injury being a predictor of future load carriage injury, the conditioning stage must move beyond general occupational conditioning to include load carriage-specific conditioning to make the soldier physically resilient against future injury and confident of their capability. By synthesizing evidence from the latest research in load carriage, this narrative review provides pragmatic considerations and guidelines for optimizing soldier load carriage capability following injury.

## 1. Introduction

From ‘Marius’s mules’ of the Roman legions [1] to the ‘grunts’ of the American infantry in Vietnam [2], soldiers have been required to physically carry heavy loads into combat [3]. These loads were, and are, imparted by the stores, equipment and weapon systems that soldiers carry into missions to provide sustainment (e.g., food and water stores), capability (e.g., communications equipment), and lethality (e.g., ammunition and weapon systems) [4]. With soldiers required to carry these loads across a variety of terrain types (e.g., urban roads, jungle terrain, sandy deserts, or beaches) and over a variety of terrain grades (ranging from flat ground to mountainous terrain) in austere conditions that can range from extreme heat to extreme cold [5,6,7,8,9,10,11,12,13,14], the combined weights of these loads can reach in excess of 45 kg [4,15]. Furthermore, history suggests that these loads will potentially increase further regardless of the advent of technology [3,16] and currently shows no signs of decreasing [17]. Given these load weights, it is not surprising that, while intending to increase elements of a soldier’s capability, these loads have altered battle tactics [18], reduced army size [18], and resulted in soldier deaths in previous conflicts [19]. Specifically, load carriage has been found to increase workload whether standing, walking or running [20,21,22,23,24,25,26,27,28,29,30,31,32,33,34,35,36], reduce cardiorespiratory capability [37,38,39,40], mobility [41,42,43], marksmanship [41,44,45,46], grenade throw ability [41], attention to task and cognitive control [41,47], and military performance in general [48]. Furthermore, these loads can cause a variety of injuries [4,16,17,49,50,51,52]. One way of mitigating the negative impacts of soldier load carriage is through ensuring soldiers are physically robust enough to withstand the forces imparted by the loads they must carry [53]. This is a concept obvious to militaries since the era of the Roman Legionnaires [54] and is still a focus in modern soldiers [55]. If a soldier is injured, ensuring they are physically capable of safely carrying occupational loads when returned to full duties is even more critical, given that not only is previous injury a leading risk factor for future injury [56], but load carriage specifically is a leading mechanism for injury [57]. Adopting a holistic lens, this review will examine the rehabilitation requirements of an injured soldier who may normally be required to undertake load carriage tasks, through three progressive stages: initial treatment, reconditioning, and occupational conditioning to develop physical resilience against injury (PRAI) and ensure the individual is physically capable and confident for return to their role.

## 2. Load Carriage Injuries

**Figure 1:**
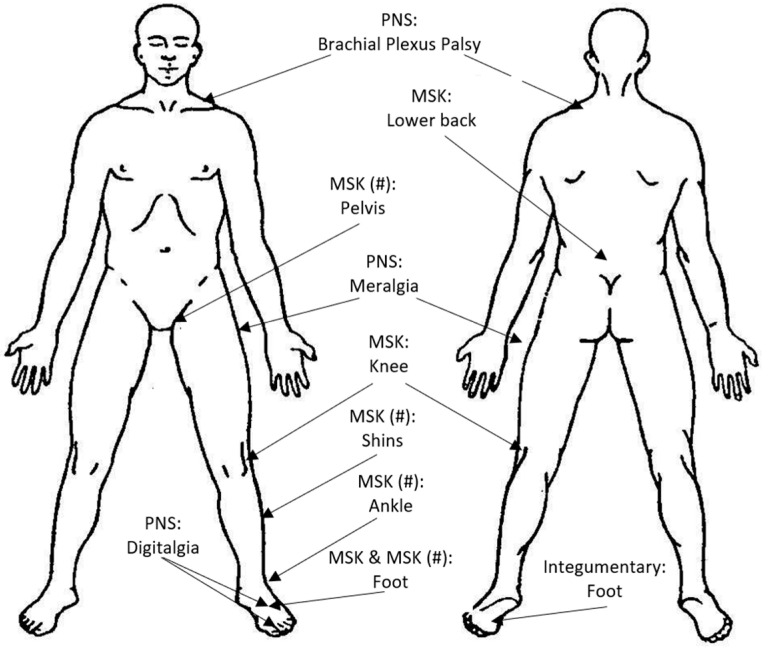
Anatomical mapping of common load carriage injuries. Legend: PNS = Peripheral Nervous System; MSK = Musculoskeletal System; MSK (#) = Musculoskeletal System Stress Fractures.

Across the career spectrum, from trainees [49,58,59,60,61] through to qualified soldiers [59,62,63,64,65,66] and those serving in Special Forces [67,68,69,70,71,72], military personnel commonly suffer injuries as a result of their service. These injuries are widely varied in nature and causes. Generally, the lower limbs are the leading body sites of injury, with musculoskeletal injuries (notably sprains and strains) the leading type of injuries [58,60,61,62,63,67,68,69,72]. Injured soldiers need to be made physically robust enough to safely return to full duties and undertake required tasks, which in the majority of cases is inclusive of load carriage. Injuries associated with load carriage itself can range from injuries to the integumentary system (e.g., blisters) and peripheral nervous system (e.g., brachial plexus palsy, meralgia paresthetica) to musculoskeletal injuries (e.g., stress fractures, muscular strains, ligamentous sprains) [3,50,51,73,74,75,76,77]. Injury data drawn from specific load carriage events [78,79] and longitudinal studies [73,75] suggest that, when body sites are grouped, the lower limbs are the leading site of musculoskeletal injuries, with the knee the most common specific lower limb site [78,79,80]. When body sites are not grouped, the lower back is often the leading site of injury [73]. An overview of load carriage injuries and associated systems is shown in Figure 1.

### 2.1. Integumentary System

Foot blistering is a frequently occurring type of integumentary system injury and is generally caused by shearing forces within the epidermis [81]. These types of injuries can occur during load carriage marches [79,80], with the risk increasing as backpack loads increase [80] due to increases in the shearing forces. Other risk factors that warrant consideration include ethnicity, smokeless tobacco use, and age [79,81]. While foot blisters may not be of great concern during load carriage conditioning, blisters may cause the carrier to alter their gait, adding load and increasing injury risk to other musculoskeletal systems as ‘hot spots’ (areas of hyperemia and precursors to blisters) develop [82]. In more extreme instances, blisters can become infected [81,82], further complicating the member’s recovery.

### 2.2. Peripheral Nervous System

Load carriage marches have been associated with neuropathies to the lower (digitalgia and meralgia) and upper (brachial plexus palsy) limbs [51,83,84,85]. Digitalgia (or digital paresthetica) presents as numbness and altered sensations (e.g., burning feeling) in the foot and toes and is postulated to be due to direct compression of the nerves innervating the feet and toes [51,85]. Potential causes of this type of compression neuropathy that should be considered during a patient’s PRAI conditioning include overuse or fatigue of lower leg muscles [86,87] and poor fitting boots [88,89].

Meralgia presents as numbness and weakness on the anterolateral aspect of the thigh [50] and is postulated to be caused by entrapment and compression of the lateral femoral cutaneous nerve [88,90,91]. Potential causes include overly tight waist belts (from backpacks or harness webbing) [88] or prolonged wearing of body armor, typically in a seated position where the armor rests on the wearer’s thighs [90]. Given the influence of equipment on this condition, consideration should be given to inspecting the member’s load carriage equipment fit during the initial stages of load carriage PRAI conditioning, with particular attention to modifying or adapting equipment placing pressure on the anterolateral thigh during sitting or load carriage.

Brachial plexus palsy (also known as rucksack or backpack palsy) presents as weakness, numbness, and pain in the upper limb [51,92,93,94,95]. Postulated causes are traction or compression of the brachial plexus nerve routes caused by backpack straps compressing the region of the upper trunk of the brachial plexus [92,94,96,97]. The risk of a brachial plexus palsy injury could be found to increase in soldiers wearing backpacks without a frame, heavy packs without a waist belt or not wearing the waist belts attached to the backpack [98,99]. As such, PRAI conditioning for load carriage should ensure that the backpack, if worn, has a frame and the waist belt is fastened correctly.

### 2.3. The Musculoskeletal System

The musculoskeletal system can suffer a variety of injuries during and following a load carriage event. The injuries range from soft tissue injuries (e.g., ligamentous sprains) to hard tissue injuries (e.g., skeletal stress fractures). Common sites for soft tissue injuries include the back, knee, ankle and foot [73,78,79,80], while common sites for stress fractures include the pelvis, shin (tibial shaft and condyles), and foot [100,101,102,103,104].

With greater force transmission through the lower limbs during load carriage [105] and the lower limbs being the leading site of injury in military populations in general, it is not surprising that the lower limbs are the leading aggregated site of injuries reported during and following a load carriage event. Typically, these lower limb injuries are to the knee, ankle, and foot, although patterns may vary between male and female soldiers, with male soldiers suffering more ankle injuries than female soldiers and female soldiers suffering more foot injuries [106] and injuries to the pelvis [107] than male soldiers. When body sites are not grouped, the lower back tends to present as the leading specific site of injury [73] and while male and female soldiers may suffer similar rates of lower back injuries overall, female soldiers tend to experience more severe low back injuries (RR = 2.40, 95% CI 0.98 to 5.88) [106].

Stress fractures are of particular concern given their long recovery periods and sometimes serious long-term consequences. A common cause of stress fractures is the repetitive overloading of the skeletal system to the point bone regeneration is outpaced by bone breakdown in the bone remodeling process [94,108,109]. Given the repetitive stress etiology and degree of tissue damage, stress fractures generally present with long and protracted recovery periods [104,107,108,110,111]. During the recovery and conditioning phases, the rates of recovery of different tissues must be considered when designing the conditioning and PRAI-specific conditioning approaches. For example, an aerobic system conditioning session that follows a load carriage session conducted the day previous could be conducted on a bicycle or in a pool to reduce skeletal load and impact and provide an ‘orthopedic’ holiday [110].

Understanding injuries specifically associated with load carriage is of importance, as it is through understanding the underlying mechanisms that injured personnel can safely move through the stages of their clinical pathway, from rehabilitation to reconditioning to PRAI conditioning. For example, female soldiers, in general, are more susceptible to pelvic stress fractures due to over striding [107]. As such, ensuring female soldiers and shorter male soldiers do not overstride when marching alone (reconditioning phase) or in a group (conditioning phase) is of importance, especially following musculoskeletal injuries to the pelvis, and load weight rather than speed of march may be preferential to control exercise intensity early in the conditioning period. As another example, while ankle injuries are less common than knee injuries during load carriage [73], they are more common in male soldiers undertaking load carriage than in female soldiers [106]. Thus, a male soldier recovering from a severe ankle injury may be more susceptible to an ankle reinjury than a female soldier when carrying loads. Following a back injury, adding intensity factors like speed and terrain may be a preferable as initial means of increasing intensity during load carriage conditioning than adding load weight, especially for lighter female soldiers who would be carrying greater relative loads and are at risk of a more severe back injury [106]. Furthermore, this understanding can ensure that the job-specific conditioning itself is designed to make the soldier PRAI through inclusion of specific load carriage conditioning rather than just applying a generic conditioning program. For example, with donning and doffing load carriage equipment (e.g., packs), a known cause of load carriage associated injury [73], safe and correct techniques to manually handling soldier packs can be taught and enforced.

## 3. The Initial Treatment Stage

Soldiers are known to underreport injuries [70,71,112,113,114], self-treat injuries [115,116,117], or delay treatment [118,119], with some injury surveillance systems failing to capture injured personnel [58]. As such, encouraging early reporting and commencement of treatment is of importance and may not only speed up recovery and return to duties but also prevent maladaptive coping strategies, pain becoming chronic and other long-term adverse outcomes including disability and impacts on mental health [118]. Noting that the initial treatment will be medical (e.g., potential medication or surgery) and allied health (e.g., physiotherapy) in nature, factors negatively impacting load carriage fitness warrant consideration, as these factors will impact the reconditioning and PRAI conditioning processes.

Two primary factors are loss of aerobic fitness and loss of relative strength through a combination of decreases in absolute strength and increased body mass associated with reduced physical activity. As will be discussed in the next section, aerobic fitness is a critical element of fitness required for load carriage [120,121] and therefore approaches to minimising aerobic fitness loss during the initial treatment period are important. For a soldier with a lower limb injury, for example, who may have difficulty with lower limb load bearing activities like walking and running, suspended deep or shallow water running or cycling, may also be of use in maintaining aerobic fitness if the nature of the injury allows (e.g., absence of surgical wounds). Alternatively, upper body boxing or an ‘arm grinder’ may be of use. If partial weight-supported positions are allowed and if the injured structure can be loaded, stationary bicycling or rowing can be used. Depending on the nature of the injury, time available, and resources, these sessions can be conducted in short-duration exercise bouts. For example, high-intensity intermittent training (HIIT), involving near maximal effort at an intensity of ≥80% maximal heart rate completing short intervals lasting ≤ 30 s with a low total training volume (≤5 min), has been found to increase aerobic fitness [122,123].

Basic strength training would also be of importance, noting that this would not be the stage in which to try to increase the soldier’s strength but rather a stage in which efforts should be made to prevent strength loss. These exercises could be generic squat, deadlift, bench press, and loaded chin up exercises [121] at training intensities and volumes known to elicit strength adaptations (e.g., loads of 80%+ of 1 Repetition Maximum (RM)/6–8 repetitions [124]). Likewise, if time is limited, training for strength and hypertrophy could be facilitated with a repetition range of 6–15 RM to a total of 4 sets per week for each major muscle group preferably using compound bilateral movements [125].

Given relative, as opposed to absolute, strength is more strongly associated with load carriage capability [126], minimising unwanted weight gain requires dedicated attention, as the soldier may reduce their incidental physical activity and thereby increase their body mass [127].

## 4. The Reconditioning Stage

Once the soldier is ready to return to activity and progressively rebuild their capacity, consideration should be given to reconditioning the soldier, both in terms of increasing the resilience of the injured tissues [128] and regaining fitness lost through incidental deconditioning [129,130]. Elements of this reconditioning should include types of physical activity associated with load carriage, these being aerobic conditioning and resistance training. Research by Robinson et al. [121] notes the importance of aerobic fitness in load carriage performance as does work by Feigel et al. [120]. Likewise, given strength is in most [121,126,131], but not all [120], cases associated with load carriage performance, relative strength training in particular should form part of the reconditioning program. With absolute strength also important for tasks that may be conducted during a load carriage task (e.g., victim drag [126]), strength training focused on increasing absolute strength in conjunction with body weight management will increase relative strength concomitantly. Of note, a volume of evidence suggests that a combination of both aerobic fitness and strength training is needed to improve load carriage performance rather than training either element of fitness alone [4,53,132,133].

As the soldier may be returning to unrestricted duties at this stage, prevention of program-induced cumulative overload (PICO) [134] and particularly limiting training to the prescribed program [135] is important if reinjury risk is to be mitigated. PICO arises as result of additional training loads occurring within or being added to the standard physical training program by other tasks that occur during the soldiers training. Such additional tasks can include, for example, military drill, weapons training, obstacle course training, defensive and offensive operations activities (e.g., fire and movement), and similar occupational tasks [134]. Spending two hours on a drill square performing unscheduled military drill or marching all around the barracks to different duties, for example, would also add unanticipated skeletal loading to a soldier recovering from stress fractures, who may also be completing a return to running program. Research has shown that just moving around the barracks and attending and completing daily tasks can see recruits cover distances of up to 17,000 steps per day [136], highlighting the potential scale of incidental loads added to personnel. Performing additional activity on top of that prescribed (e.g., unit sports training, going for a personal run) may likewise increase the training load on the soldier and negatively impact their recovery, as such additional activities have been shown to increase injury risk in soldiers [135].

While training load monitoring may be considered to help identify and mitigate this excessive load and prevent further injury, practical application in tactical populations may be problematic due to the number of variables associated with injuries in these populations (e.g., smoking, previous injury, sleep and circadian rhythm disruption, PICO, etc.) and lack of equipment [137]. However, consideration should still be given to load monitoring approaches where possible. Acute/Chronic Workload Ratios, present as one viable option noting, however, that the ranges of 0.8–1.3 are based on sporting populations rather than military personnel [137]. Monitoring of Heart Rate Variability (HRV) may also be of use [137,138]. However, the correct HRV metric must be selected and considered against what the data collection device’s fidelity and capability [139]. Finally, as treatment may be multidisciplinary, who would be collecting and monitoring this data capture requires consideration as does the time required to download, interpret, and action the data. In addition to monitoring load, optimizing recovery following days of high load may assist in mitigating reinjury during this stage. Case-dependent recovery strategies could include, but are not limited to, cold water immersion therapy [140], contrast bathing [141], cryotherapy [142], and compression garments [143]. In addition, advice on optimizing sleep and nutrition (via referral to a nutritionist) may be of benefit.

Finally, any fear or anxiety in anticipation of returning to duty is a normal protective response and should be identified (e.g., Return to Duty Readiness Questionnaire [144]) and addressed, as this may negatively impact progression during the reconditioning stage and thus transfer to the conditioning stage.

## 5. The Occupational Conditioning Stage

With load carriage tasks a source of injury risk, the conditioning stage needs to be targeted with a PRAI focus. Following the principle of specificity and specific adaptation to imposed demands [145], it is not surprising that the ability to complete a load carriage activity has been found to be the biggest predictor of future load carriage performance [121]. As such, during the conditioning phase, it is essential that load carriage tasks be introduced if they are to be a requirement of the soldier when returning to duty. This would include progressing to carrying a weapon and/or load carriage in the hands, on the head, on the thigh, and in other expected ways, as these factors impact mechanical loading on different anatomical sites and alter energy costs [146]. However, as discussed above, load carriage itself is a source of injury [75] and as such, a load carriage conditioning program must be progressive, manipulating volume and intensity variables in a carefully structured way. Research in this field suggests an optimal frequency of load carriage sessions to be one session every 7–14 days [147,148] and that increasing the frequency to more than this may increase injury risk without increasing performance [74]. Regarding duration or distance (i.e., volume), these should be progressed to meet the requirement of the job task (e.g., 15 km march if this is part of the soldier’s physical employment requirements). Intensity is varied through manipulation of load weight, speed of movement, and the terrain grade and type [4]. These intensity variables have all been found to separately increase the intensity of a load carriage event [4,149,150,151,152,153]. For example, increases in speed of march from 5.0 to 5.5 km/h are similar to increasing load weight by 10 kg in terms of energy costs, as is increasing the grade of the terrain by 1% [4]. Effective manipulation of these variables (i.e., load weight, speed of march and terrain) to optimise conditioning has been shown to increase load carriage performance and decrease injuries in military personnel [49].

These variables (i.e., volume and intensity) should be applied with consideration of the injury from which the soldier is recovering, and injury/symptom response closely monitored. For example, if the mechanism of the soldier’s injury was overuse, shorter, more intense load carriage sessions may be more beneficial than longer slower events. For soldiers who have suffered injury due to the mechanism of overstriding, increasing load weight and marching at their own stride length (i.e., not marching ‘in step’ with other soldiers) may be appropriate. For soldier’s who have suffered a joint injury from an axial loading mechanism (e.g., medial meniscal tear [154], lumbar disc injury [155]), using marching speed and increasing the terrain grade (as opposed to changing terrain type) may be a more suitable means of increasing intensity than adding load weight. For these soldiers, joint distraction (the gentle pulling apart the bones in a joint to create space) activities, like chin ups and aquatic training in deep water, may further facilitate recovery.

The combination of resistance and aerobic training, which has been associated with improvements in load carriage performance [121,147,148], should be continued and extended beyond reconditioning to conditioning designed to increase these elements of fitness. With poor aerobic and musculoskeletal fitness associated with increased injury risk [156,157,158,159], increasing these elements of fitness will not only further improve load carriage performance but also increase the soldier’s PRAI. Considering this, one downstream impact of a load carriage session on other elements of the conditioning program is the subsequent reduction in neuromuscular function, which may last 48–72 h after a load carriage activity [160,161,162]. For example, immediately following a load carriage march, lower leg power as measured by a vertical jump has been found to decrease [162]. Research by Leyk et al. [161] found reductions in soldier grip strength over a longer period—72 h—after a load carriage session involving a stretcher carry. With vertical jump power associated with sprinting [163,164] and simulated military tasks [165,166], and with grip strength associated with deadlift performance [167,168] and victim drag performance [169,170,171,172], fatigue following load carriage sessions may impact the performance of other conditioning exercises and tasks performed up to 3 days later.

While this paper has focused on the physical aspects of load carriage, several other considerations warrant attention, including placement of load around the body, concurrent military tasks, and concurrent cognitive requirements and challenges imparted by load carriage. Noting that soldiers may be required to carry loads on both the front and rear of their torse, on their head, in their hands, on their thigh, and on their feet [4,150], and that load placement can influence the energy costs of conducting a load carriage task and induce local muscle fatigue, this specificity needs to be progressively introduced during the PRAI conditioning. Soldiers often complete tasks (as opposed to simply marching) while wearing and carrying load [173,174,175,176] and may also assume other body positions (e.g., taking a knee) while loaded. As such, integrating the load carriage conditioning into other military skills (e.g., stretcher carry, stores move, fire and manoeuvre, etc.) and adopting other body positions (e.g., kneeling unsupported fire position) should form part of the conditioning. Given that load carriage can negatively impact cognitive demands, like attention-to-task [13], the load carriage conditioning needs to progress beyond simply carrying the load and walking to include cognitive tasks. Remembering items randomly placed along a given route to be recalled later (e.g., Kims games [177]) or continuous alphanumeric sequences (e.g., n-back task [178]) (activities know to increase cognitive demand [179,180]), serves as examples of how cognitive demand can be incorporated in the load carriage conditioning program. Similarly, if soldiers are exhibiting anxiety about the risk of reinjury or pain during their return to load carriage and consciously or unconsciously adopting protective behaviours, strategies like graded exposure and desensitisation [181] may be adopted. Such strategies enable soldiers to progressively (gradually) build the demands and complexity of load carriage in real-world scenarios while being reassured at each step by the fact the progression has not reinjured them. Tolerance to manageable levels of anxiety is encouraged and developed during this process until ultimately the anxiety dissipates, along with protective behaviours. As part of the process, some level of pain or discomfort may be normalised as the soldier comes to realise that some discomfort is common as they progress through their return to load carriage, is not indicative of further injury, and will often reduce with time and practice. In these ways, the soldier’s confidence and capability in load carriage can be effectively rebuilt. Finally, a coordinated, multidisciplinary approach that includes the clinical team, military physical training instructors (or equivalent), and the chain of command should be involved so as to provide a coordinated multidisciplinary approach with the roles and responsibilities within the team established early in the rehabilitation process. Effective communication and collaboration strategies are essential, particularly during transition periods, such as the handover of the soldier from clinical care to physical training. Through regular team meetings, notably with the soldiers, the medical, physical training and command elements for a safe return to load carriage tasks tailored to both individual and operational demands can be optimised.

## 6. Conclusions

Dedicated load carriage rehabilitation is required for soldiers who are required to carry loads as part of their occupation. This load carriage rehabilitation should span all three stages of the soldier’s return-to-work pathway from minimizing loss of critical elements of fitness during the initial injury stage to reconditioning these fitness elements during the reconditioning stage to commencing load carriage-specific conditioning with a focus on making the soldier physically resilient against injury during the conditioning stage. Manipulation of volume (session frequency and length) and intensity (load weight, speed of march and terrain grade) should be considered in light of the soldier’s presenting injury and known load carriage-associated risks along with other factors which may indirectly impact their recovery (e.g., fear of returning to duty, doing unplanned or unconsidered additional activity, etc.) should also be considered.

## Data Availability

All data informing this review is publicly available.

## Abbreviations

The following abbreviations are used in this manuscript:

PICO	Program-Induced Cumulative Overload
PRAI	Physical Resilience Against Injury
